# Clinical application and improvement of a CNN‐based autosegmentation model for clinical target volumes in cervical cancer radiotherapy

**DOI:** 10.1002/acm2.13440

**Published:** 2021-10-13

**Authors:** Yankui Chang, Zhi Wang, Zhao Peng, Jieping Zhou, Yifei Pi, X. George Xu, Xi Pei

**Affiliations:** ^1^ Institute of Nuclear Medical Physics University of Science and Technology of China Hefei China; ^2^ Radiation Oncology Department First Affiliated Hospital of Anhui Medical University Hefei China; ^3^ Radiation Oncology Department First Affiliated Hospital of University of Science and Technology of China Hefei China; ^4^ Radiation Oncology Department First Affiliated Hospital of Zhengzhou University Zhengzhou China; ^5^ Anhui Wisdom Technology Co., Ltd. Hefei Anhui China

**Keywords:** adaptive improvement, autosegmentation, clinical target volumes, deep learning

## Abstract

**Objective:**

Clinical target volume (CTV) autosegmentation for cervical cancer is desirable for radiation therapy. Data heterogeneity and interobserver variability (IOV) limit the clinical adaptability of such methods. The adaptive method is proposed to improve the adaptability of CNN‐based autosegmentation of CTV contours in cervical cancer.

**Methods:**

This study included 400 cervical cancer treatment planning cases with CTV delineated by radiation oncologists from three hospitals. The datasets were divided into five subdatasets (80 cases each). The cases in datasets 1, 2, and 3 were delineated by physicians A, B, and C, respectively. The cases in datasets 4 and 5 were delineated by multiple physicians. Dataset 1 was divided into training (50 cases), validation (10 cases), and testing (20 cases) cohorts, and they were used to construct the pretrained model. Datasets 2–5 were regarded as host datasets to evaluate the accuracy of the pretrained model. In the adaptive process, the pretrained model was fine‐tuned to measure improvements by gradually adding more training cases selected from the host datasets. The accuracy of the autosegmentation model on each host dataset was evaluated using the corresponding test cases. The Dice similarity coefficient (DSC) and 95% Hausdorff distance (HD_95) were used to evaluate the accuracy.

**Results:**

Before and after adaptive improvements, the average DSC values on the host datasets were 0.818 versus 0.882, 0.763 versus 0.810, 0.727 versus 0.772, and 0.679 versus 0.789, which are improvements of 7.82%, 6.16%, 6.19%, and 16.05%, respectively. The average HD_95 values were 11.143 mm versus 6.853 mm, 22.402 mm versus 14.076 mm, 28.145 mm versus 16.437 mm, and 33.034 mm versus 16.441 mm, which are improvements of 37.94%, 37.17%, 41.60%, and 50.23%, respectively.

**Conclusion:**

The proposed method improved the adaptability of the CNN‐based autosegmentation model when applied to host datasets.

## 1 INTRODUCTION

Cervical cancer is one of the most common malignant tumors in the female genital tract worldwide,^1^ and its treatments include radiotherapy, chemotherapy, surgery and comprehensive medicine. Radiotherapy plays an important role in the treatment of cervical cancer and can be used at all stages with good curative effects. Accurate delineation of clinical target volume (CTV) contours in cervical cancer is time consuming and labor intensive for radiation oncologists, and observer variability exists among radiation oncologists and the unclearly defined tumor‐to‐normal tissue boundary. Autosegmentation methods are needed to alleviate oncologists’ workloads and increase the consistency of delineation.

Previously, many studies have proposed methods for autosegmentation, including atlas‐based methods, convolutional neural network (CNN)‐based methods, level‐set methods, and morphological methods. Atlas‐based methods and CNN‐based methods are the most widely used methods, and studies have shown that CNN‐based methods^2^, ^3^, ^4^, ^5^ can obtain greater accuracy and better efficiency than Atlas‐based methods.^6^, ^7^, ^8^ CNN‐based methods have made great achievements in medical image segmentation. Long et al.^9^ proposed a fully convolutional network (FCN) for semantic segmentation, which is the benchmark for image segmentation. Based on FCN, Ronneberger et al.^10^ proposed U‐Net for biomedical image segmentation, and Milletari et al.^11^ proposed V‐shaped FCN for 3D medical image segmentation. Xing et al.^12^ proposed super pixel‐based and boundary‐sensitive CNN (SBBS‐CNN), which can take full advantage of the rich special contextual information, for liver segmentation. Liu et al.^13^ modified U‐Net for automatic segmentation of organs at risk (OARs) in cervical cancer and obtained promising results. Men et al.^14^ proposed a deep dilated CNN (DDCNN) model to segment CTV and OARs in rectal cancer.

However, it is difficult to obtain high accuracy when applying autosegmentation models to host datasets (that have little relevance to the training data) directly because of data heterogeneity and interobserver variability (IOV). Wong et al.^15^ expressed concerns about the applicability of autosegmentation models to external data because the validation datasets are very closely related to the training datasets in many studies. Li et al.^16^ compared artificial intelligence (AI) contouring with the contouring of eight qualified radiation oncologists for delineating gross tumor volume (GTV) of nasopharyngeal carcinoma by MRI, which proved the IOV among radiation oncologists and compared the difference between AI and human experts. Liu et al.^17^ proposed MS‐Net for improving prostate segmentation with heterogeneous MRI data and consistently enhancing the performance across all datasets. Although previous works pointed out the problem of data heterogeneity and IOV, there have been few studies on how to improve the adaptability of autosegmentation models that were trained with less relative training data.

To that end, this study aims to research an adaptive improved method for applying an autosegmentation model on host datasets. We modified the 3D Res‐U‐Net model for CTV autosegmentation of cervical cancer and applied the pretrained model at multiple sites. Dataset 1 was used to establish the pretrained model, and datasets 2–5 were collected for measuring and observing the improvements.

In this paper, we introduce the patient datasets in Section 2.1, the network architecture and data preprocessing in Section 2.2, and the concrete experimental process in Section 2.3. Then, we present the experimental results in Section 3 and discuss the experimental results and related research in Section 4.

## 2 METHODS

### 2.1 Patient datasets

A total of 400 cervical cancer cases from three hospitals, including 240 cases from the First Affiliated Hospital of Anhui Medical University in China (hospital A), 80 cases from the First Affiliated Hospital of University of Science and Technology of China (hospital B), and 80 cases from the First Affiliated Hospital of Zhengzhou University (hospital C), were collected in this study. All cases were divided into 5 datasets (80 cases in each dataset; datasets 1–3 are from hospital A, dataset 4 is from hospital B, and dataset 5 is from hospital C). The cases in datasets 1, 2, 4, and 5 were collected during clinical radiation therapy between January 2019 and May 2020. Dataset 1 and dataset 2 were delineated by physicians A and B, respectively, while datasets 4 and 5 were delineated by multiple physicians. The manual delineation of the cervical cancer CTV was conducted in accordance with the guidelines of by the Radiation Therapy Oncology Group (RTOG),^18^ which starts from the bifurcation of the common iliac artery and includes the primary tumor, uterus, appendix, part of the vagina (the upper half or two‐thirds of the vagina according to the primary tumor), and pelvic lymph nodes (common iliac, external iliac, internal iliac, obturator, and presacral).

**FIGURE 1:**
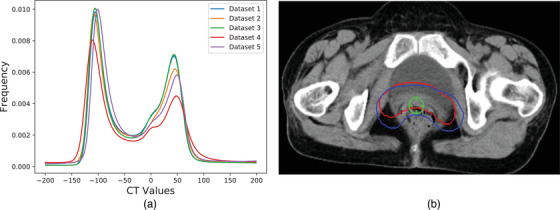
(a) Frequency distribution histograms of the CT values for the multisite datasets. The threshold range is (–200, 200). We only counted the CT values where the CTV was located according to the delineation results of the physician. To prevent negative effects caused by using particular cases, we selected 20 cases as one group for statistical analysis. (b) CTV delineations of two different physicians for the same patient. Red lines: manual contours delineated by physician A; Blue lines: manual contours delineated by physician C

Previous studies showed^19^, ^20^, ^21^, ^22^, ^23^ that the same physician can have different manual contours at different times due to fatigue and other factors, which means that a physician may be more consistent in delineating CTV contours at shorter intervals. In view of this, we collected 80 cases from hospital A for dataset 3, and they were delineated by a physician over 2 successive months. The details of the CT information in the different datasets are listed in Table 1. The frequency distribution histograms of the CT values from multiple datasets are shown in Figure 1a, and CTV delineations of two different physicians are shown in Figure 1b. Table 1 and Figure 1a show that heterogeneity and variability exist among the different datasets, Figure 1b shows that the IOV exists among the different physicians.

**TABLE 1:** Details of the CT information in the different datasets

Dataset	Manufacturer	Slice thickness (mm)	Pixel dimension (mm)	CTV volumes (cm^3^)	Number of CTV layers^*^
Dataset 1	Siemens	5	0.789–0.977	928 ± 258	40
Dataset 2	GE	5	0.703–1.074	1069 ± 322	46
Dataset 3	Siemens	5	0.789–0.977	759 ± 149	37
Dataset 4	GE	2.5	0.977	894 ± 201	43
Dataset 5	Siemens	5	1.27	746 ± 168	41

* Number of CTV layers was counted when the slice thickness was resampled to 5 mm.

### 2.2 Data preprocessing and model training

In this study, special data preprocessing was implemented for CTV autosegmentation of cervical cancer. First, the original data are truncated by the threshold (–200, 200), values below –200 are set as –200 and values above 200 are set as 200. Next, the CT values are normalized to (0, 1) to increase the generalization ability of the model. Then, the resolutions of the images are resampled to 1 mm × 1 mm × 5 mm due to inconsistent resolutions in the datasets. To focus on the segmentation of CTV and reduce the irrelevant information in the background, we precut the CT images to a size of 288 × 288.

**FIGURE 2:**
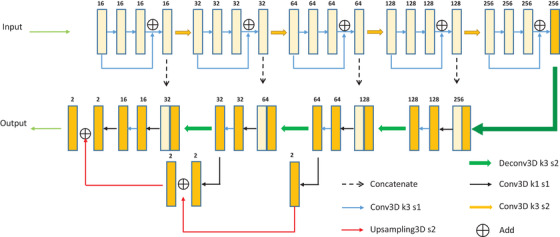
Overall architecture of our proposed 3D convolutional neural network

The proposed network in this study is shown in Figure 2 and consists of an encoder that extracts features from data and a decoder that conducts the segmentation task. The network is based on 3D U‐Net and combines many advanced techniques, including skip connections, residual modules and deep supervision. In the final three segmentation blocks, a 1 × 1 × 1 convolution layer is used to map the feature tensor to the probability tensor before all results are merged by the upsampling operation to enhance the precision of segmentation results. The model accuracy was validated in our previous work.^24^, ^25^ During training, data augmentation was used to alleviate overfitting. Specifically, CT patches with a size of 192 × 192 × 64 were randomly cropped from precut CT images and then fed to the network for training.

The architecture of the model remained the same throughout the whole experiment. This model training was finished in Python with TensorFlow using Nvidia Geforce RTX 2080Ti GPUs (11 G). The batch size was set to 1 due to the limitation of GPU memory. The Adam optimizer ($\beta_{1}=0.9,\beta_{2}=0.999,lr=0.0005$) and the instance normalization were used, and the weighted Dice similarity coefficient (DSC) was selected as the loss function. The leaky rectified linear unit was used as the convolution activation layer and the SoftMax activation was used to output a probability of every voxel. It should be emphasized that early stopping was employed for model training. The learning rate was initialized as $5.0\times 10^{-4}$ and was divided by 10 when the validation loss did not significantly decrease in 50 successive epochs. If the validation loss failed to decrease in 80 successive epochs, the model training was stopped automatically. The purpose of using early stopping in this experiment was to make the model stop at the optimal level.

### 2.3 Experiment

**FIGURE 3:**
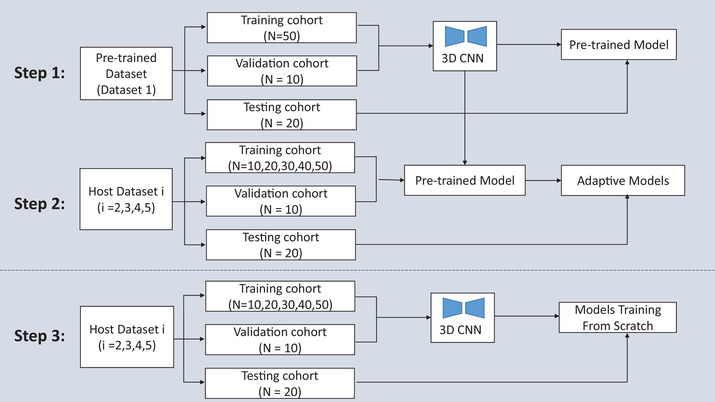
Pipeline of the experiment

As mentioned above, although the five datasets were focused on CTV contours in cervical cancer, there were differences between them due to data heterogeneity and IOV. In addition, different physicians at the same hospital or the same physician at different times could produce different manual contour results. In this study, we plan to evaluate the accuracy of a pretrained autosegmentation model on host datasets and study the adaptive improvement in the autosegmentation model. The pipeline of the experiment is shown in Figure 3 and includes step 1 (establishing the pretrained model and evaluating the model accuracy), step 2 (fine‐tuning the model on host datasets based on the pretrained model), and step 3 (training on the same datasets as in step 2 but from scratch).

First, we implemented a 3D CNN for delineating CTV of cervical cancer on dataset 1, which was divided into training, validation, and testing cohorts of 50, 10, and 20 cases. After model training and validation, the cases in the testing cohort were used to evaluate the performance of the model. The trained model was considered the pretrained model for step 2 to check the accuracy of the pretrained model on host datasets and research the adaptive improvement in clinical applications.

Subsequently, we studied the adaptive improvement of the pretrained model on datasets 2–5. The cases in host dataset *i* (*i* = 2, 3, 4, 5) were divided into training, validation and testing cohorts with 50, 10, and 20 cases, respectively. Different from step 1, the 50 cases in dataset *i* were not used for training every time and were randomly divided into 5 subgroups with 10 cases in each subgroup. In the process of model fine‐tuning, the 20 cases in the testing cohort were fixed, but the number of cases in the training cohort gradually increased from 10 to 50 (10, 20, 30, 40, and 50). According to the number of cases in the training cohort, the fine‐tuning models were clustered into five categories. To reduce potential bias, all possible combinations of the five subgroups were considered in this study when the number of training cases was 10, 20, 30, and 40. We performed sixfold cross validation when the number of training cases was 50. The performance of the fine‐tuning model was evaluated by the average performance of all models inside each category (the same number of the fine‐tuning cases belong to the same category, including 10, 20, 30, 40, and 50 cases, respectively).

Finally, step 3 was used to compare and analyze the difference between training with pretrained model and training from scratch. Other than the use of the pretrained model, the data grouping and model structure were exactly the same as those in step 2. The models in step 3 were all trained from scratch, and the corresponding testing cohort was used to test the accuracy of these models. Step 3 could be considered the baseline compared to step 2.

### 2.4 Evaluation metrics

The DSC and 95% Hausdorff distance (HD_95) values were used to evaluate the accuracy of the autosegmentation model. The DSC is defined as follows^26^:

(1) $$DSC=\frac{2\left|A\cap B\right|}{\left|A\right|+\left|B\right|},$$

where *A* denotes the autosegmentation contours and *B* denotes the ground truth contours delineated by the physicians in our study. A larger DSC corresponds to a higher accuracy of autosegmentation model. The DSC ranges from 0 to 1, with the latter value indicating perfect performance.

The HD is defined as follows:

(2) HD(A,B)=max(h(A,B),h(B,A)),

(3) $$h\left(A,B\right)=\max_{b\in B}\left(\min_{a\in A}\left\|a-b\right\|\right),$$

where h(*A*, *B*) is the greatest of all the distances from a point in *A* to the closest point in *B*. A smaller value usually represents better segmentation accuracy. The HD_95 value represents the largest surface‐to‐surface separation among the closest 95% of surface points.

## 3 RESULTS

**FIGURE 4:**
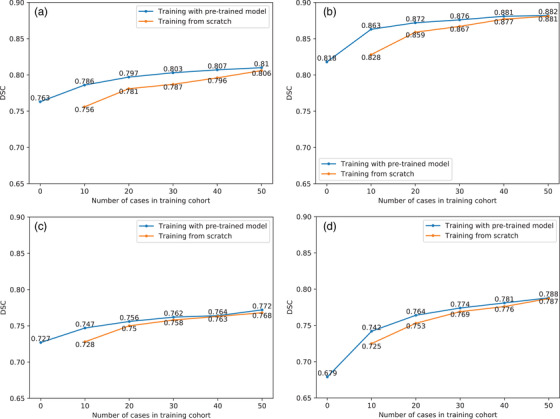
DSC values of the test cases on the host datasets. (a) Dataset 2; (b) dataset 3; (c) dataset 4; (d) dataset 5

The average DSC value of the test cases of the pretrained model (dataset 1) was 0.852±0.023, and the DSC values of the test cases on the host datasets are summarized in Table 2 and displayed in Figure 4. When the pretrained model was directly applied to the test cases of datasets 2–5, the average DSC values were 0.763, 0.818, 0.727, and 0.679, respectively. After fine‐tuning with 50 cases in the training cohort, the average DSC values were 0.810, 0.882, 0.772, and 0.788 for datasets 2–5, which were increases of 6.16%, 7.82%, 6.19%, and 16.05%, respectively. With the increase in the number of training cases, the difference between training with pretrained model and training from scratch gradually decreased. After training with 50 cases, there were almost no differences (*p* > 0.05) for datasets 2–5 when trained with or without pretrained model (0.810 vs. 0.806, 0.882 vs. 0.881, 0.772 vs. 0.768, and 0.788 vs. 0.787).

**TABLE 2:** DSC values of the test cases on the host datasets

	Dataset 2		Dataset 3		Dataset 4		Dataset 5	
Number of cases in the training cohort	TWP	TFS	TWP	TFS	TWP	TFS	TWP	TFS
0	0.763 ± 0.045		0.818 ± 0.028		0.727 ± 0.044		0.679 ± 0.060	
10	0.786 ± 0.050	0.756 ± 0.054	0.863 ± 0.032	0.828 ± 0.050	0.747 ± 0.051	0.728 ± 0.062	0.742 ± 0.064	0.725 ± 0.066
20	0.797 ± 0.047	0.781 ± 0.049	0.872 ± 0.028	0.859 ± 0.035	0.756 ± 0.047	0.750 ± 0.050	0.764 ± 0.054	0.753 ± 0.059
30	0.803 ± 0.043	0.787 ± 0.049	0.876 ± 0.025	0.867 ± 0.031	0.762 ± 0.046	0.758 ± 0.046	0.774 ± 0.047	0.769 ± 0.054
40	0.807 ± 0.044	0.796 ± 0.044	0.881 ± 0.023	0.877 ± 0.026	0.764 ± 0.043	0.763 ± 0.043	0.781 ± 0.045	0.776 ± 0.051
50	0.810 ± 0.043	0.806 ± 0.043	0.882 ± 0.023	0.881 ± 0.025	0.772 ± 0.040	0.768 ± 0.039	0.788 ± 0.045	0.787 ± 0.050
P1 value	0.002		<0.001		0.002		<0.001	
P2 value	0.797		0.838		0.752		0.890	

TWP, training with the pretrained model; TFS, training from scratch.

P1 value: pretrained model vs. TWP; P2 value: TWP vs. TFS.

**FIGURE 5:**
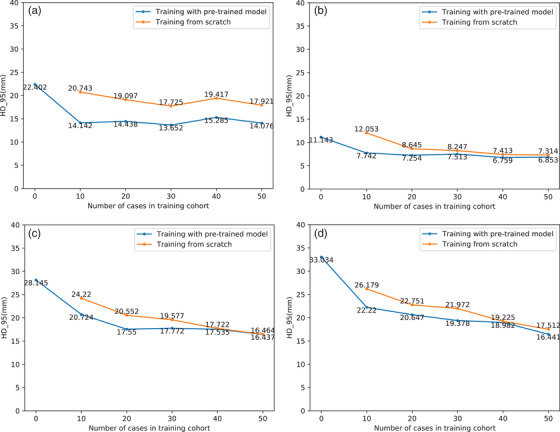
HD_95 values (mm) of the test cases on the host datasets. (a) Dataset 2; (b) dataset 3; (c) dataset 4; (d) dataset 5

The average HD_95 value of the test cases of the pretrained model (dataset 1) was 10.028 ± 3.595 mm, and the HD_95 values (mm) of the test cases on the host datasets are summarized in Table 3 and displayed in Figure 5. For datasets 2–5, the average HD_95 values decreased from 22.402, 11.143, 28.145, and 33.034 mm to 14.076, 6.853, 16.437, and 16.441 mm, respectively, which are decreases of 37.17%, 38.50%, 41.60%, and 50.23%. Similar to the DSC analysis, there was almost no difference (*p* > 0.05) whether the pretrained model was used after training with 50 cases in the training cohort. The average HD_95 values were 14.076 mm versus 17.921 mm, 6.853 mm versus 7.314 mm, 16.437 mm versus 16.464 mm, and 16.441 mm versus 17.512 mm, respectively.

**TABLE 3:** HD_95 values (mm) of the test cases on the host datasets

	Dataset 2		Dataset 3		Dataset 4		Dataset 5	
Number of cases in the training cohort	TWP	TFS	TWP	TFS	TWP	TFS	TWP	TFS
0	22.402		11.143		28.145		33.034	
10	14.142 ± 1.398	20.743 ± 5.514	7.742 ± 0.185	12.053 ± 1.225	20.724 ± 1.975	24.220 ± 3.874	22.220 ± 3.572	26.179 ± 4.527
20	14.438 ± 2.252	19.097 ± 5.488	7.254 ± 0.252	8.645 ± 0.726	17.550 ± 1.104	20.552 ± 2.460	20.647 ± 3.560	22.751 ± 4.417
30	13.652 ± 1.579	17.725 ± 3.395	7.513 ± 1.333	8.247 ± 1.010	17.772 ± 1.606	19.577 ± 2.344	19.378 ± 2.924	21.972 ± 1.937
40	15.285 ± 2.556	19.417 ± 2.261	6.759 ± 0.413	7.413 ± 0.488	17.535 ± 0.694	17.722 ± 0.973	18.982 ± 4.132	19.225 ± 3.385
50	14.076 ± 0.545	17.921 ± 2.859	6.853 ± 0.279	7.314 ± 0.109	16.437 ± 0.587	16.464 ± 0.960	16.441 ± 0.967	17.512 ± 2.206
P1	<0.001		0.003		0.012		0.001	
P2	0.408		0.180		0.255		0.388	

TWP, training with the pretrained model; TFS, training from scratch.

P1 value: pretrained model vs. TWP; P2 value: TWP vs. TFS.

**FIGURE 6:**
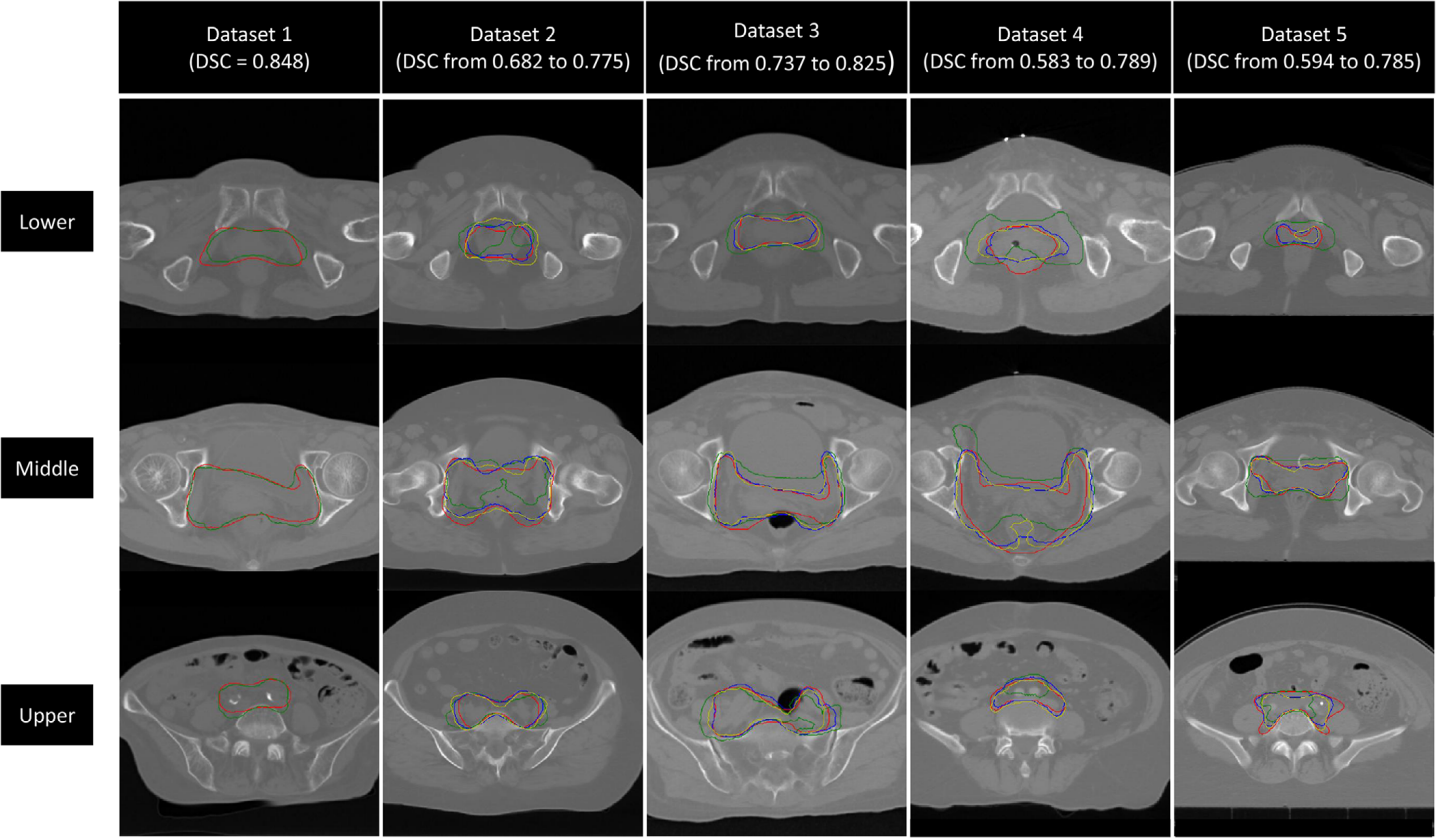
CTV contour results in different datasets. Red lines: manual contours; Green lines: autosegmentation from the original pretrained model; Blue lines: autosegmentation from the adaptive model; Yellow lines: autosegmentation from the model training from scratch

The DSCs distribution for datasets 2–5 were summarized in Table 4 and the CTV contour results in different datasets were shown in Figure 6. The improvement of the accuracy was obvious in the adaptive process. The above results show that there was almost no difference between training with pretrained model and training from scratch when the number of cases in the training cohort was sufficient. However, there was a difference in training times, which are recorded and summarized in Table 5. As shown in Table 5, the maximum time‐savings percentage is 68.8% in the training case situations, and the minimum is 19.5%, indicating that the efficiency of training with pretrained model is substantially higher than that of training from scratch.

**TABLE 4:** Dices similarity coefficients (DSCs) distribution

DSC ranges	Dataset 2			Dataset 3			Dataset 4			Dataset 5		
	PM	TWP	TFS	PM	TWP	TFS	PM	TWP	TFS	PM	TWP	TFS
<0.6	0	0	0	0	0	0	2	0	0	2	0	0
0.6–0.7	1	0	0	0	0	0	5	0	0	11	1	1
0.7–0.8	16	8	8	4	0	0	13	15	15	6	11	12
>0.8	3	12	12	16	20	20	0	5	5	1	8	7

PM, pretrained model; TWP, training with the pretrained model; TFS, training from scratch.

**TABLE 5:** Average training times (hours) of the different models

	Dataset 2			Dataset 3			Dataset 4			Dataset 5		
Number of cases in the training cohort	TWP	TFS	Time saving percent	TWP	TFS	Time saving percent	TWP	TFS	Time saving percent	TWP	TFS	Time‐savings percentage
10	0.45	0.95	52.6%	0.50	1.60	68.8%	0.75	1.00	25.0%	0.70	1.30	46.2%
20	0.65	1.50	56.7%	0.82	1.85	55.7%	1.00	2.15	53.5%	1.25	1.87	33.2%
30	1.00	2.70	63.0%	1.13	2.60	56.5%	1.40	2.40	41.7%	1.70	2.50	32.0%
40	1.50	2.45	38.8%	1.40	3.30	57.6%	1.90	2.90	34.5%	2.10	3.00	30.0%
50	1.80	2.50	28.0%	2.60	4.20	38.1%	2.20	3.00	26.7%	3.30	4.10	19.5%

TWP, training with the pretrained model; TFS, training from scratch.

## 4 DISCUSSION

In recent years, deep learning‐based methods have been widely used in medical image segmentation. To make this technology serve more people, many commercial deep learning‐based autosegmentation software programs have emerged, such as Limbus Contour,^27^ AiContour of Linking Med,^28^ and DeepViewer.^29^ When applied in clinical practice, the accuracy of autosegmentation has decreased to varying degrees due to data heterogeneity and IOV. In this study, we constructed a 3D CNN for automatically delineating CTV of cervical cancer and researched the adaptive improvement when applying the autosegmentation model to host datasets. In addition, we also compared the difference between training with pretrained model and training from scratch in accuracy and efficiency.

We found that the pretrained model had higher accuracy in the test cases that used pretrained dataset (dataset 1, DSC: 0.852) than in the test cases that used the host datasets (datasets 2–5; DSC: 0.763, 0.818, 0.727, and 0.679) when applying the pretrained model on datasets 1–5 directly. The DSC gap between the best accuracy (dataset 1) and the worst accuracy (dataset 5) was 0.173, which is a major problem for the generalization of autosegmentation model. The more relevant the training cases used for pretrained model were, the higher the accuracy was. As mentioned above, all cases in dataset 1 were from the same hospital and delineated by the same physician, so the test cases in dataset 1 were closely related to the training cases; hence, the influence of data heterogeneity and IOV could be ignored. Compared to the cases in dataset 1, the test cases in datasets 2–3 were also from hospital *A* but were manually delineated by two different physicians, so there were slight differences in the training cases compared to those in dataset 1. The test cases in datasets 4–5 were from other hospitals and delineated by multiple physicians, so there existed more difference among those test cases and training cases. In the comparison among datasets 2–5, we found that the differences in data from the same hospital (dataset 1 vs. datasets 2–3) were less than the differences in data from different hospitals (dataset 1 vs. datasets 4–5).

In the adaptive improvement process, the accuracy of autosegmentation model on the host datasets was improved to varying degrees. The average DSC values improved by 6.16%, 7.82%, 6.19%, and 16.05%, and the HD_95 values improved by 37.17%, 38.50%, 41.60%, and 50.23%, respectively. Ultimately, the accuracy of autosegmentation model depended more on the consistency within the dataset. The improved autosegmentation model achieved the highest accuracy on dataset 3, even higher than the original accuracy on dataset 1 (DSC: 0.882 vs. 0.852). Considering the difference between dataset 1 and dataset 3, the cases in dataset 3 were delineated by the same physician over 2 successive months; hence, the delineation consistency in dataset 3 was higher than that in dataset 1. The cases in dataset 2 were also delineated by the same physician, and the lower accuracy showed that the delineation “habits” of this physician were easily affected at different times. In addition, the accuracies on datasets 4–5 were lower than those of datasets 2–3, indicating that IOV within the datasets affected the improvement in the autosegmentation model to a great degree. No matter how long the autosegmentation model was trained, it was difficult to balance the differences within the dataset. In future clinical applications, we can establish different autosegmentation models for different physicians, and these models can learn the respective delineation “habits” of different physicians with high accuracy.

To verify the function of the pretrained model, two parallel experiments were implemented in this study and included training with pretrained model and training from scratch. Except for whether the pretrained model was used, the other parameters were exactly the same. When the number of training cases was small, training with pretrained model had higher precision and efficiency. With the increase in the number of training cases, the difference between training with pretrained model and training from scratch gradually decreased. When 50 cases were used for training, there was almost no difference in accuracy. In terms of both accuracy and efficiency, training with pretrained model was as accurate as training from scratch but with higher computational efficiency. For example, in dataset 3, training with pretrained model on 30 cases obtained a comparable accuracy as training from scratch on 50 cases (DSC: 0.876 vs. 0.881); however, it required less time (1.13 h vs. 4.20 h). Loading the pretrained model could make the autosegmentation model adaptive to host datasets with higher accuracy and efficiency.

Several limitations of this study should be noted. First, more representative datasets should be collected. Second, the research object should not be limited to CTV, and different research objects may obtain different results. We plan to solve the above limitations in future work.

In conclusion, we investigated the use of a 3D CNN model for delineating CTV of cervical cancer and researched the adaptive improvement when applying the autosegmentation model on host datasets. Our results showed that training with the pretrained model could make the autosegmentation model adaptive to host datasets with higher accuracy and efficiency, which could reduce the negative impacts of data heterogeneity and IOV. It is promising for deep learning methods to be able to serve more people with higher accuracy.

## AUTHOR CONTRIBUTIONS

Paper idea: Yankui Chang, Xi Pei, and X. George Xu. The source of datasets: Zhi Wang, Jieping Zhou, and Yifei Pi. Autosegmentation model: Yankui Chang and Zhao Peng. Writing of the paper: Yankui Chang, Zhao Peng, Xi Pei, and X. George Xu.

## CONFLICT OF INTEREST

The authors declare no conflict of interest.
